# Crystal Structure of the HSV-1 Fc Receptor Bound to Fc Reveals a Mechanism for Antibody Bipolar Bridging

**DOI:** 10.1371/journal.pbio.0040148

**Published:** 2006-05-02

**Authors:** Elizabeth R Sprague, Chu Wang, David Baker, Pamela J Bjorkman

**Affiliations:** **1**Division of Biology, California Institute of Technology, Pasadena, California, United States of America; **2**Department of Biochemistry, University of Washington, Seattle, Washington, United States of America; **3**Howard Hughes Medical Institute, University of Washington, Seattle, Washington, United States of America; **4**Howard Hughes Medical Institute, California Institute of Technology, Pasadena, California United States of America; Washington University School of MedicineUnited States of America

## Abstract

Herpes simplex virus type-1 expresses a heterodimeric Fc receptor, gE-gI, on the surfaces of virions and infected cells that binds the Fc region of host immunoglobulin G and is implicated in the cell-to-cell spread of virus. gE-gI binds immunoglobulin G at the basic pH of the cell surface and releases it at the acidic pH of lysosomes, consistent with a role in facilitating the degradation of antiviral antibodies. Here we identify the C-terminal domain of the gE ectodomain (CgE) as the minimal Fc-binding domain and present a 1.78-Å CgE structure. A 5-Å gE-gI/Fc crystal structure, which was independently verified by a theoretical prediction method, reveals that CgE binds Fc at the C _H_2-C _H_3 interface, the binding site for several mammalian and bacterial Fc-binding proteins. The structure identifies interface histidines that may confer pH-dependent binding and regions of CgE implicated in cell-to-cell spread of virus. The ternary organization of the gE-gI/Fc complex is compatible with antibody bipolar bridging, which can interfere with the antiviral immune response.

## Abstract 2

The structure of the C-terminal domain of Herpes simplex virus type-1 Fc receptor identifies interface regions implicated in cell-to-cell spread of virus.

## Introduction

Herpes simplex virus type-1 (HSV-1) has evolved several strategies to escape detection by the host's immune system, including the expression of an Fc receptor (FcR) called gE-gI that is found on the surface of virions and infected cells [ 1– 3]. gE-gI binds the Fc region of immunoglobulin G (IgG), likely interfering with antibody-mediated viral clearance [ 4]. Previous studies suggested that anti-HSV IgG antibodies participate in antibody bipolar bridging, whereby an antibody molecule simultaneously binds to gE-gI with its Fc region and to a specific HSV-antigen (e.g., gC or gD) with its Fab arms [ 5– 8]. Antibody bipolar bridging has been shown to protect the virus and infected cells from IgG-mediated immune responses, such as antibody- and complement-dependent neutralization [ 6], antibody-dependent cell-mediated cytotoxicity [ 5], and granulocyte attachment [ 8]. Experiments in HSV-1–infected mice comparing the effectiveness of human anti-HSV IgG versus nonimmune IgG or murine anti-HSV IgG (which does not bind gE-gI) have provided support for the importance of antibody bipolar bridging mediated by gE-gI [ 7]. gE-gI has also been implicated in the cell-to-cell spread of HSV, although the relationship between IgG binding and cell-to-cell spread remains unclear [ 9, 10].

HSV-1 gE-gI is a heterodimer composed of two type I membrane glycoproteins: gE, a 530-residue protein including a ˜401-residue extracellular region followed by a predicted single transmembrane helix and a ˜106-residue C-terminal cytoplasmic tail; and gI, a 370-residue protein including a ˜248-residue extracellular portion followed by a predicted single transmembrane helix, and a ˜94-residue C-terminal cytoplasmic tail. The cytoplasmic tails of both gE and gI contain potential endocytosis motifs, and studies of gE and gI in HSV-1 and other alphaherpesviruses have demonstrated that gE, gI, and gE-gI undergo endocytosis and recycling [ 11]. The discovery that gE-gI binding to Fc is sharply pH dependent, such that binding is observed at neutral or slightly basic pH but not at acidic pH, suggests that IgG bound by cell-surface gE-gI would dissociate if endocytosed together with gE-gI into intracellular vesicles [ 12]. In addition, the pH dependence of the gE-gI/Fc interaction allows speculation that the participation of gE-gI in antibody bipolar bridging plays an active role in clearing the cell surface of host IgG and of viral antigens, whereby endocytosis of HSV antigens bound to gE-gI–associated anti-HSV IgG is followed by dissociation of the IgG-antigen complexes in degradative compartments such as lysosomes.

Several studies have identified regions of gE, gI, and Fc that are critical to the gE-gI and gE-gI/IgG interactions. Although the gE-gI heterodimer is required for high-affinity binding of monomeric IgG, gE alone is a low-affinity FcR that binds isolated Fc [ 12] as well as IgG in immune complexes, such as IgG-coated erythrocytes [ 1, 13]. Because isolated gI binds neither free IgG nor IgG in immune complexes [ 13], it is unlikely to contain a significant Fc binding interface. The Fc binding region on gE has been localized to the C-terminal domain of the gE ectodomain (CgE) [ 14, 15], whereas the N-terminal domain of gE (NgE) associates with gI, forming a complex that does not bind to IgG [ 16]. The region of the gE-gI heterodimer that is important for cell-to-cell spread of HSV has also been mapped to CgE [ 17, 18].

The gE-gI-binding site on IgG has been localized to the Fc C _H_2-C _H_3 interdomain hinge, a “hot spot” that serves as the binding site for several other mammalian and bacterial Fc-binding proteins [ 19, 20]. Residues critical to the interaction were identified in binding studies comparing the affinities of human IgG subtypes for gE-gI, which found that gE-gI binds to IgG1, IgG2, and IgG4 with similar affinities (equilibrium dissociation constant [K _D_] ˜40–400 nM), but does not bind several IgG3 allotypes or an IgG4 mutant in which His435 was changed to an arginine [ 21, 22]. These results implicate a role for Fc His435, which is an arginine in most human IgG3 allotypes, and are consistent with a previous suggestion that gE-gI and protein A have overlapping binding sites at the C _H_2-C _H_3 domain interface of Fc [ 23]. Additional studies using a nonbinding Fc mutant (nbFc), which contains six point mutations in the C _H_2-C _H_3 hinge, confirmed the gE-gI binding site on Fc and established the stoichiometry as two gE-gI heterodimers per one Fc dimer (2:1) [ 12], analogous to the binding stoichiometries for all known FcR/Fcγ interactions that involve the Fc C _H_2-C _H_3 domain interface [ 19, 24– 28].

In order to study the interaction between gE-gI and Fc in more detail, we initiated structural studies of gE, gE-gI, and a gE-gI/Fc complex. gE-gI heterodimers are resistant to crystallization, but we were able to determine structures of a gE fragment and a gE-gI complex with Fc. Here we describe the 1.78-Å crystal structure of CgE, demonstrate that CgE binds to Fc, and present a low-resolution structure of a gE-gI/Fc complex. The binding mode observed for Fc and the CgE portion of gE-gI in the gE-gI/Fc crystal structure was verified by an independent theoretical prediction of the CgE interaction with Fc. The gE-gI/Fc model resulting from prediction and crystallographic methods is consistent with biochemical data characterizing the interaction, provides insight into the molecular basis for the observed pH dependence of the gE-gI/Fc interaction, and allows mapping of CgE residues important for IgG binding and cell-to-cell spread. In addition, the orientation of the gE-gI/IgG complex on a membrane, as predicted from the complex crystal structure, demonstrates that antibody bipolar bridging can occur on the surface of an infected cell or virus.

## Results

### CgE Binds Fc in the Absence of Other gE or gI Domains

**Figure 1:**
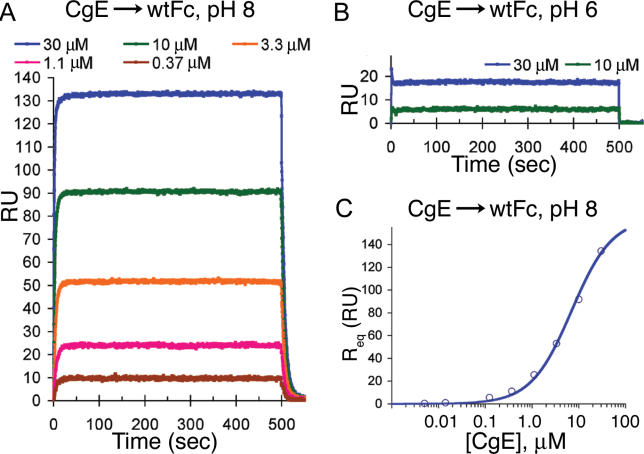
Binding of CgE to Fc at pH 8 and pH 6 (A and B) Sensorgrams collected from the indicated concentrations of CgE injected at pH 8 (A) or pH 6 (B) over a flow cell coupled with wtFc. (C) Plot of equilibrium-binding response (R _eq_) versus concentration of CgE at pH 8 derived from biosensor data shown in (A). The best-fit binding curve to the experimental data points (open circles) is shown as a continuous line.

Previous studies of the gE-gI/IgG and gE/IgG interactions localized the Fc-binding region of gE to residues 235–380, which are contained within CgE [ 14, 15]. To determine whether isolated CgE (gE residues 213–390, where residue 1 is the first residue of the hydrophobic leader peptide in the immature gE and residue 420 is the first residue of the predicted transmembrane region) binds Fc and to measure the affinity of the interaction, we performed a surface plasmon resonance assay using two forms of recombinant Fc derived from human IgG1: wild-type Fc (wtFc) and nbFc, which contains six point mutations in the C _H_2-C _H_3 linker that abrogate binding to gE-gI (Met252 to Gly, Ile253 to Gly, His310 to Glu, His433 to Glu, His435 to Glu, and Tyr436 to Ala) [ 12]. The Fc proteins were immobilized on the surface of a biosensor chip, and the binding of CgE was assayed at pH 8 and pH 6. Significant binding of CgE to nbFc was not observed under any conditions (unpublished data), but CgE binds wtFc with a K _D_ of ˜7 μM at pH 8 ( Figure 1A and 1C). The relatively low affinity of the CgE/Fc interaction likely explains why a CgE/Fc binding interaction was not detectable by coimmunoprecipitation analyses [ 16]. The affinity of CgE for Fc is similar to that determined for the interaction of gE with Fc at pH 8 (K _D_ ˜30 μM), but ˜50-fold weaker than the affinity of the gE-gI/Fc interaction, which has K _D_ values of 340 nM and 930 nM for the first and second binding sites on wtFc, respectively [ 12]. As previously observed for the interaction of gE-gI with Fc [ 12], the binding of CgE to Fc is much weaker at pH 6 than at pH 8 ( Figure 1A and 1B). In combination with previous data, these results suggest that the gI chain contributes to the gE-gI interaction with Fc either directly or through stabilizing the binding conformation of gE, that NgE does not contribute to Fc binding, and that at least some of the pH dependence of the gE-gI interaction with Fc can be attributed to the CgE/Fc interface.

### Structure of the Fc-Binding Domain of gE

**Figure 2:**
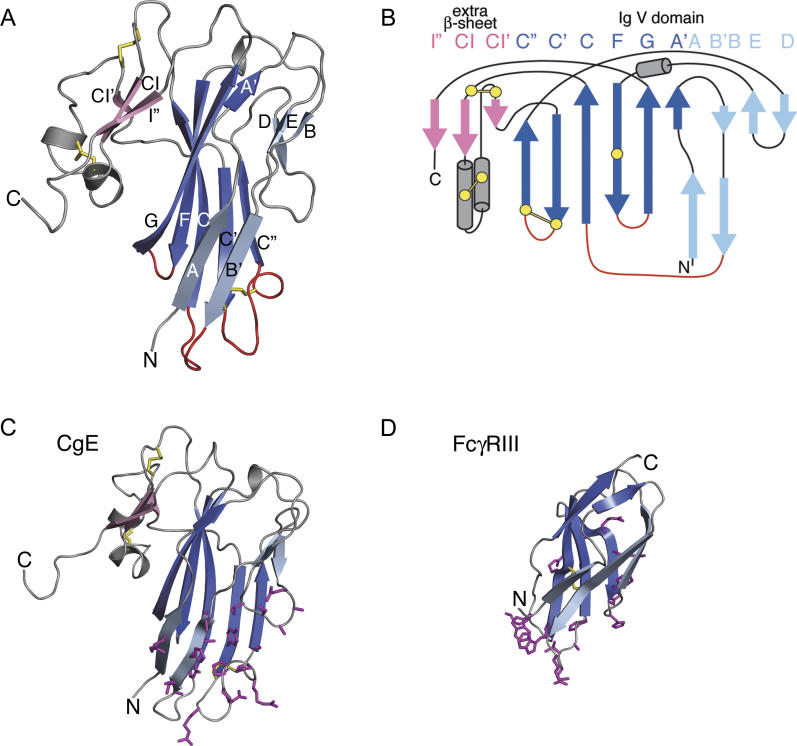
Structure of CgE (A) Ribbon diagram of the CgE structure. β-strands D, E, B, B′, and A are light blue, strands A′,G, F, C, C′, and C′′ are dark blue, strands CI′, CI, and I′′ are pink, α-helical regions are gray, disulfide bonds are yellow, and the three CDR loops (as defined in Ig V domain structures) are red. All figures depicting protein structures were generated with PyMOL (The PyMOL Molecular Graphics System, http://www.pymol.org). (B) CgE topology diagram. β-strands are shown as arrows (colored as in [A]), and helices are shown as gray cylinders. Cysteines are shown as yellow circles with disulfide bonds indicated by yellow lines between paired cysteines. (C) Ribbon diagram of CgE with Fc-binding residues highlighted. β-sheets are colored as in (A) (D E B B′ A, light blue; A′ G F C C′ C′′, dark blue; and CI′ CI I′′, pink), and side chains in the predicted Fc-binding interface (see text) are in magenta. Disulfide bonds are in yellow. (D) Ribbon diagram of FcγRIII with Fc-binding residues highlighted. β-sheets for FcγRIII (pdb entry 1e4j) are colored similar to (C) (E B A, light blue; and A′ G F C C′, dark blue), and side chains in the Fc-binding interface [ 44] are in magenta. Disulfide bonds are in yellow.

The structure of CgE was determined to 1.78 Å by multiple anomalous dispersion using a crystal of selenomethionine (SeMet)–substituted CgE ( Table S1). CgE is composed primarily of 14 β-strands organized into three β-sheets, two of which pack together in a manner resembling an Ig variable (V) fold ( Figure 2A and 2B). The first and second β-sheets in CgE, which correspond to their counterparts in Ig V domains, contain strands A, B, B′, E, and D, and strands A′, G, F, C, C′, and C′′, respectively. A third sheet, which is not found in Ig V domains or other Ig superfamily members, is a parallel β-sheet containing strands CI, CI′, and I′′ (strands denoted with “I” are part of the “inserted” β-sheet, with strands CI and CI′ proceeding strand C) ( Figure 2A and 2B). The third β-sheet packs against the backside of the A′GFCC′C′′ sheet on the opposite face from the DEBB′A sheet ( Figure 2A).

A 3-D search of the protein database using the DALI server [ 29] identified the coxsackie virus and adenovirus receptor domain 1 (pdb entry 1eaj) and a mouse Fv (pdb entry 1mfa) as two of its closest structural neighbors, with root mean square deviations of 2.6 Å over 108 Cα atoms and 2.8 Å over 106 Cα atoms, respectively ( Figure S1). The structural similarity between CgE and its structural homologs does not include the third β-sheet found in CgE. In addition, CgE contains disulfide bonds that are not typically found in Ig superfamily folds: Cys271–Cys297, which joins the N-terminal ends of the CI and CI′ strands; Cys280–Cys289, between residues within the helices near the CI and CI′ strands; and Cys314–Cys323, between residues within the complementarity-determining region 2 (CDR2) loop ( Figure 2A and 2B). The CgE fold lacks the disulfide bond that joins the A′GFCC′C′′ and the DEBB′A sheets in the canonical Ig fold, with CgE containing a cysteine in strand F (Cys359) but a valine in the complementary position in strand B (Val244). However, the conserved tryptophan normally found in strand C adjacent to the intersheet disulfide bond is present in CgE (Trp262). Further structural differences include the extension of strands C, C′, and C′′ as well as the A′–B and C′–C′′ loops, and the shortening of strands D and E as compared to a representative Ig V domain ( Figure S1A), which leads to a more open organization of the first two β-sheets (sheets DEBB′A and A′GFCC′C′′). In addition, the DEBB′A sheet is a “split” β-sheet composed of two smaller β-structures (strands DEB and B′A) linked by the split B/B′ strand ( Figure 2A and 2B).

### Comparison with Other FcRs

Other IgG receptors of known structure, which include the mammalian FcγR proteins found on the surface of immune cells, also belong to the Ig superfamily (reviewed in [ 30]). Structural similarity between CgE and host FcγRs was suggested based on a 38-residue region of sequence similarity (46% amino acid identity between human FcγRII residues 109–154 and gE residues 322–359) and the spacing between gE residues Cys323 and Cys359, which is similar to the typical spacing between cysteines in the disulfide bond linking strands B and F in Ig folds [ 14]. However, CgE has an Ig V folding topology, whereas host Ig superfamily FcγRs are C2-type Ig domains (reviewed in [ 30]). As a result, the 38-residue region of sequence similarity does not adopt a similar structure in CgE and the FcγRs: CgE residues 322–359 encompass strands C′′, D, E, and F, whereas strands C′′ and D are absent in C2-type Ig domains, and the predicted Cys323-Cys359 disulfide bond [ 14] is not present; instead Cys323 is in a disulfide bond with Cys314, and Cys359 is not bonded to another cysteine ( Figure 2C and 2D).

The only other FcR of known structure to adopt an Ig V domain fold is the polymeric Ig receptor (pIgR), which contains an N-terminal V-like domain that binds to polymeric IgA and IgM [ 31]. A comparison of the CgE and pIgR domain 1 structures does not reveal significant similarity beyond a region of common folding topology (unpublished data). Differences between the pIgR and CgE Ig V folds include the positions of the CDR loops, the predicted Fc-binding sites (described for CgE below), the overall size, with CgE being larger due to its extended strands C, C′, and C′′, and the third β-sheet in CgE, which is absent in pIgR domain 1.

### Crystal Structure of a gE-gI/Fc Complex

Attempts to crystallize a gE-gI heterodimer or gE alone were unsuccessful (ERS, unpublished data), thus we concentrated our efforts on obtaining a structure of a gE-gI/Fc complex. Crystals of gE-gI bound to Fc were large, easily reproduced, and had an ideal morphology, but diffracted weakly to ˜5-Å resolution. The complex crystals could not be improved by changing the gE-gI or Fc construct or by manipulating the crystals (summarized in Materials and Methods). A 5.0-Å native data set was collected from the best of several hundred crystals ( Table S2) and used for molecular replacement with the IgG Fc (pdb entry 1dn2) and CgE structures as search models. A molecular replacement solution was obtained when the Fc was positioned first followed by two CgE molecules, one bound to each Fc chain. Although the volume of the asymmetric unit could accommodate one to three gE-gI/Fc complexes [ 32], further searches did not reveal additional Fc or CgE molecules, suggesting that the gE-gI/Fc crystals contain a single 2:1 complex of gE-gI/Fc. The resulting unusually high solvent content (79%) may explain the limited resolution of the crystals.

**Figure 3:**
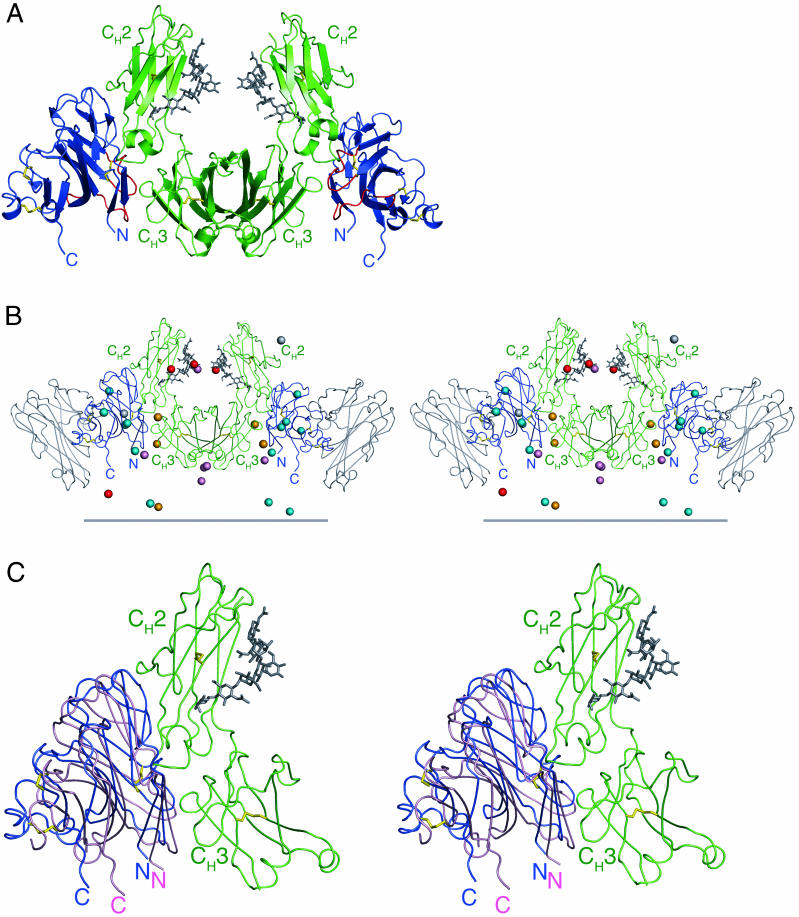
Experimentally Determined and Predicted Structures of CgE Bound to Fc (A) Ribbon diagram of the top molecular replacement solution for a 2:1 CgE/Fc complex. CgE is shown in blue with the CDR loops highlighted in red, and Fc is shown in green. Disulfide bonds are shown in yellow. (B) Stereo view of the location of bound heavy atoms and SeMet residues superimposed on the CgE/Fc model derived by molecular replacement. CgE is blue and Fc is green. The predicted position of the cell membrane (see text) is indicated by a gray line. A CgE molecule related by crystallographic symmetry is shown in gray to demonstrate that the remaining portions of the gE-gI/Fc structure (NgE and gI) cannot occupy this location. The heavy-atoms and SeMet residues are shown as spheres and colored by atom type (gray, mercury; red, tungsten; pink, iridium; orange, platinum; and teal, SeMet). The heavy-atom and SeMet positions identified by Solve [ 56] were expanded by the crystallographic symmetry operators and translated to the symmetry-equivalent positions that are closet to the CgE/Fc model. (C) Stereo superposition of the crystallographically determined CgE/Fc complex and the complex predicted with RosettaDock [ 34] using the structures of CgE and Fc. A half complex (one CgE and one chain of the Fc dimer) is shown with Fc in green, the CgE as positioned in the crystal structure in blue, and the CgE as positioned by the docking prediction in pink. The root mean square deviation between the Cα atoms of the Rosetta-positioned CgE molecule and each of the CgE molecules in the molecular replacement solution is 3.9 Å, with the alignment being the best in the regions of CgE that are closest to the CgE/Fc interface and poorest in the regions that are more distant from the binding site.

The molecular replacement solution positions a CgE molecule near the C _H_2-C _H_3 linker on each of the Fc chains to make an approximately two-fold symmetric 2:1 gE-gI complex ( Figure 3A). The model allows sufficient space in the unit cell for gI and NgE, which are unaccounted for in the molecular replacement solution, and shows a plausible packing arrangement including contacts between crystallographically related CgE molecules. Other features of the molecular replacement solution suggest that it is correct. First, two CgE molecules are bound to each Fc dimer, in agreement with the observed stoichiometry of the gE-gI/Fc interaction [ 12]. Second, although symmetry was not imposed, the solution is nearly perfectly two-fold symmetric with the two-fold axis relating the two CgE molecules coinciding with the Fc dyad axis and each CgE molecule making similar contacts with the same region of an Fc chain. Finally, the CgE molecules are positioned to interact with the C _H_2-C _H_3 linker region of Fc, consistent with previous biochemical analyses of the gE-gI/Fc interaction [ 12, 21].

We used experimental phasing methods to independently corroborate the molecular replacement model. A screen of complex crystals that had been soaked in various heavy-atom compounds resulted in four approximately isomorphous data sets ( Table S2). An initial analysis of a KIrCl _3_ derivative identified five iridium sites located near the Fc molecule as positioned in the molecular replacement solution, four of which are related by the Fc dimer symmetry and two of which make a chemically-plausible interaction with a methionine (Met358) on each half of the Fc dimer ( Figure 3B). Phases were calculated after locating heavy atom sites for the other derivatives (PIP, EMP, and Na _2_WO _4_) and used to find the locations of 13 of 18 potential selenium-substituted methionines in a crystal of SeMet-substituted gE-gI complexed with Fc. A comparison of the identified selenium sites with the molecular replacement model showed that eight of the 13 sites mapped to the eight methionine residues in CgE ( Figure 3B). The clustering of all of the heavy atom sites around the Fc molecule provides further support for only one copy of the complex in the asymmetric unit. An overlay of the molecular replacement model with a 5-Å heavy atom–phased electron density map ( Figure S2) shows overlap of the model with electron density, including density for the *N*-linked carbohydrates attached to the C _H_2 domain of Fc, and extra density that likely corresponds to gI and NgE. Thus, experimental phasing methods using a combination of heavy-atom derivatives and SeMet-substituted crystals provides further confirmation of the molecular replacement solution and validates its use as a model for the CgE/Fc interaction in the context of the gE-gI/Fc complex.

### Independent Prediction of the CgE/Fc Interaction

A complementary, computational approach was carried out in parallel to the crystallographic work in order to model the CgE/Fc interaction independently using an improved version of RosettaDock [ 33, 34]. RosettaDock uses real-space Monte Carlo minimization on both rigid-body and side-chain degrees of freedom to identify the lowest free energy docked arrangement of two protein structures and can be guided by biological constraints when available. Docking was carried out in three steps using the CgE structure and one chain of the two-fold symmetric Fc used in the molecular replacement search. In the first step, a global search through possible rigid-body orientations of the CgE and Fc proteins was carried out with the constraint that the CgE/Fc interface contains at least one of the six residues in the consensus binding site on Fc (Met252, Ile253, Ser254, Asn434, His435, and Tyr436) [ 19]. In similar calculations on a number of other complexes [ 35], a small number of closely related models that are significantly lower in energy than others is observed, and in such cases, these low-energy models are almost always close to the correctly docked structure. The magnitude of the energy gap between the correct models and other models is correlated with the binding affinity of the interaction [ 33]. Perhaps because of the relatively low binding affinity of the CgE/Fc interaction ( Figure 1C), we did not observe a significant energy gap in this case, and hence could not confidently identify a single model as correct based on the global docking calculations alone. Instead, we proceeded by clustering the lowest-energy models based on structural similarity and further pruning high-ranked (large-sized) clusters by imposing a second constraint that CgE interacts with both the C _H_2 and C _H_3 domains of Fc, which has been observed for other interactions with the consensus Fc binding site [ 19]. In the last step, the centers of the top five clusters were subjected to rigid-body and side-chain torsion refinement, and the lowest energy models from each refinement run were selected as the final predictions. Of the five resulting models for the CgE/Fc interaction, one is similar to the crystallographic model obtained by molecular replacement and experimental phasing methods ( Figure 3C).

### Overview of the CgE/Fc Interface

The identification of the C _H_2-C _H_3 linker region of the IgG Fc as the gE-gI binding site allows comparison with other proteins that share similar binding sites on Fc. Thus, the gE-gI/Fc complex structure provides a robust starting point for understanding general features of the gE-gI interaction with Fc, despite the caveats that small conformational changes in the CgE or Fc structures and/or flexibility of the C _H_2-C _H_3 hinge cannot be addressed due to the limited resolution of the structure.

**Figure 4:**
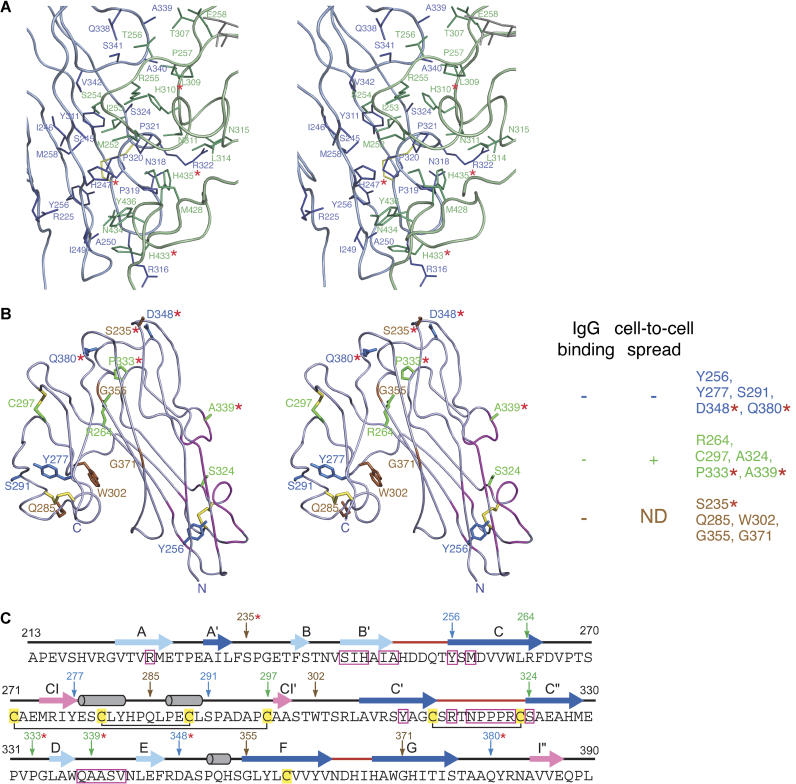
Interfaces of CgE Involved in IgG Binding and Cell-to-Cell Spread (A) A close-up of predicted side-chain interactions in the CgE/Fc interface. CgE side chains and peptide backbone are blue, Fc side chains and peptide backbone are green, and disulfide bonds are yellow. Regions above and below the plane of the interaction have been omitted for clarity. Residues are defined as interacting if one or more side-chain atoms from a residue in one protein are within 5 Å of a side-chain atom on the partner protein, which includes residues 225, 245–247, 249–250, 256, 258, 311, 316, 318–322, 324, and 338–342 on CgE and 252–258, 307, 309–311, 314–315, 382, 428, and 433–436 on Fc. Histidine residues in the interface are indicated with red asterisks. Fc residue 382 is not visible in this orientation of the interface. (B) Insertion mutants in CgE that disrupt Fc binding and/or viral spread mapped onto the CgE structure. The CgE backbone is shown in blue with regions that are predicted to be in the CgE/Fc interface shown in magenta. The side chains shown are colored according to the effect that a four- or five-residue linker inserted after them had on IgG binding and viral spread (blue, disrupt IgG binding and viral spread; green, disrupt IgG binding only; and brown, disrupt IgG binding and not tested for viral spread [ 7, 14, 17, 18, 38, 39]). Red asterisks indicate residues in surface-exposed loops. (C) Secondary structure of HSV-1 CgE (residues 213–390) mapped on the amino acid sequence. Elements of secondary structure are shown above the sequence with color-coding as in Figure 2A and 2B, and the three CDR loops (as defined in Ig V domain structures) are red. Cysteine residues are highlighted in yellow, with disulfide bonds indicated by black lines between paired cysteines. The insertion mutagenesis data described in (B) is depicted as colored arrows above the sequence with red asterisks indicating residues in surface-exposed loops. Residues that are in the predicted CgE/Fc interface based on the crystallographic data are boxed in magenta.

The complex structure shows that CgE interacts with Fc using regions of the first and second β-sheets, which correspond to the two β-sheets found in Ig V domains: strands A and B′ and the B′–C and D–E loops in the DEBB′A sheet and strands C, C′, and C′′ and the C′–C′′ loop in the A′GFCC′C′′ sheet ( Figures 2C and 4). The third β-sheet does not contact Fc and is distant from the CgE/Fc interface ( Figures 2C, 4A, and 4C). Of the CgE loops that correspond to the CDR loops in Ig V domains, only CDR2 makes extensive contacts with Fc ( Figures 2C, 4A, and 4C).

The C _H_2-C _H_3 linker region on Fc has been identified as a “hot spot” for protein–protein interactions [ 19, 20]. Crystal structures are available for Fc complexed with four other proteins, domain B1 of protein A [ 25], domain C2 of protein G [ 28], rheumatoid factor [ 24], and FcRn [ 27], and with one peptide that bind to this region [ 19]. Each of the Fc-binding proteins is different in sequence and structure; however, their binding interactions with Fc are similar, sharing a set of six contact residues on Fc that have been referred to as the consensus region [ 19, 27]. Interface interactions involving side-chain atoms of Fc residues Met252, Ile253, Ser254, Asn434, His435, and Tyr436 and the adjacent peptide backbone are found in each of the five FcR/Fc interfaces [ 19, 27], and are predicted to occur in the CgE/Fc interface ( Figure 4A). Likewise, the gE-gI/Fc structure suggests that the interface with CgE contains at least two conserved interactions with Fc that are found in three or more of the five FcR/Fc interfaces [ 19, 27]: hydrophobic packing onto Fc His435 by gE Pro319 and gE Pro321, and hydrophobic packing onto Fc Met252 and Fc Tyr436 by gE His247 ( Figure 4A). Similar to the binding interfaces for the other five proteins that bind the C _H_2-C _H_3 linker region of Fc [ 19, 20, 27], the CgE/Fc interface is primarily nonpolar with a total of ˜1800 Å ^2^ of buried surface area.

The gE-gI/Fc model is consistent with previous observations regarding the characteristics of Fc binding by gE-gI. The finding that HSV-1 gE-gI does not bind an Fc mutant containing six point mutations in the C _H_2-C _H_3 linker (Met252 to Gly, Ile253 to Gly, His310 to Glu, His433 to Glu, His435 to Glu, and Tyr436 to Ala) [ 12] that are all predicted to be in the CgE/Fc interface can be explained by both a reduction in the nonpolar interface area and an unfavorable burial of charge in a hydrophobic environment ( Figure 4A). Similarly, the lack of gE-gI binding by allotypes of human IgG3 that contain a His to Arg substitution at position 435 compared to IgG1, IgG2, and IgG4 [ 21, 22] is likely due in part to the unfavorable energetic consequences of burying an extended, charged residue in the small hydrophobic pocket formed by gE residues Pro319 and Pro321 ( Figure 4A). A comparison of the sequences of the Fc regions of the human, rabbit, and rat IgG subclasses, focusing on the Fc residues that the gE-gI/Fc model predicts to be important for Fc binding (Fc residues 252–258, 307, 309–311, 314–315, 382, 428, and 433–436), suggests a molecular basis for the observation that gE-gI binds to human and rabbit IgG, but not to rodent IgG [ 21, 36]. Many of the Fc residues in or near the interface are either identical or conservatively substituted in human, rabbit, and rat; however, differences at positions 252, 255, 258, and 382 (human/rabbit Met252, Arg255, Glu258, and Glu382 versus rat Thr/Leu/Met252, Leu/Gln255, Lys258, and Gln/Lys/Thr/Glu382) could perturb the interface such that gE-gI does not bind rat IgG.

### Mechanism of pH-Dependent Binding

Previous studies demonstrated a sharply pH-dependent interaction for HSV-1–infected cells binding to rabbit IgG [ 37] and for soluble gE-gI or gE monomers binding to human Fc [ 12], such that binding occurs at neutral or slightly basic pH but not at acidic pH. An analysis of the change in the affinity of the gE-gI/Fc complex versus pH suggested that two ionizable residues, likely histidines because they have a pK _a_ near neutral pH, participate in the pH-dependent affinity transition [ 12]. The pH-dependent binding of Fc to CgE ( Figure 1A and 1B) suggests a direct role for CgE in modulating the gE-gI/Fc interaction.

The CgE/Fc interface predicted from the gE-gI/Fc structure contains four histidine residues that are candidates for mediating the pH-dependent affinity transition: gE His247 near Fc Met252, Met428, and Tyr436; Fc His310 near gE Pro321 and Ala340; Fc His433 near gE Ile249, Ala250, Arg316; and Fc His435 near gE Pro319 and Pro320 ( Figure 4A). The residues in the vicinity of these histidines are generally nonpolar, and protonation of a histidine residue at acidic pH results in a positive charge that would be unfavorable if buried in a hydrophobic environment or near another positively charged residue. We predict that protonation of at least two of the four histidines in the interface alters the chemical properties of CgE and/or Fc such that the gE-gI/Fc interaction is disrupted at acidic pH. The histidines at Fc positions 310 and 435 appear to be more buried than those at Fc position 433 and gE position 247, suggesting that Fc His310 and Fc His435 are the two most likely histidines to cause Fc dissociation upon protonation at acidic pH. pH-dependent conformational changes in CgE and/or Fc could also disrupt binding at acidic pH. Although we cannot rule out local conformational variability that occurs as a function of pH, it seems unlikely that CgE undergoes large changes in conformation due to its relative compactness as a single domain, and arguments against pH-induced conformational changes in Fc have been made based on the similarity of Fc crystal structures at pH values between 4.1 and 8.0 [ 27].

### Structure-Based Interpretation of gE Mutagenesis Studies

To identify regions of gE involved in IgG binding or cell-to-cell spread of HSV-1, a series of four- to five-residue insertions were introduced into gE [ 7, 14, 17, 18, 38, 39]. In the absence of a 3-D structure, the effects of insertions on the local and global conformations of a protein are unpredictable. However, the structures of CgE and a gE-gI/Fc complex reported here can be used to identify which insertions are likely to disrupt the CgE structure and which insertions are likely to affect function.

**Figure 5:**
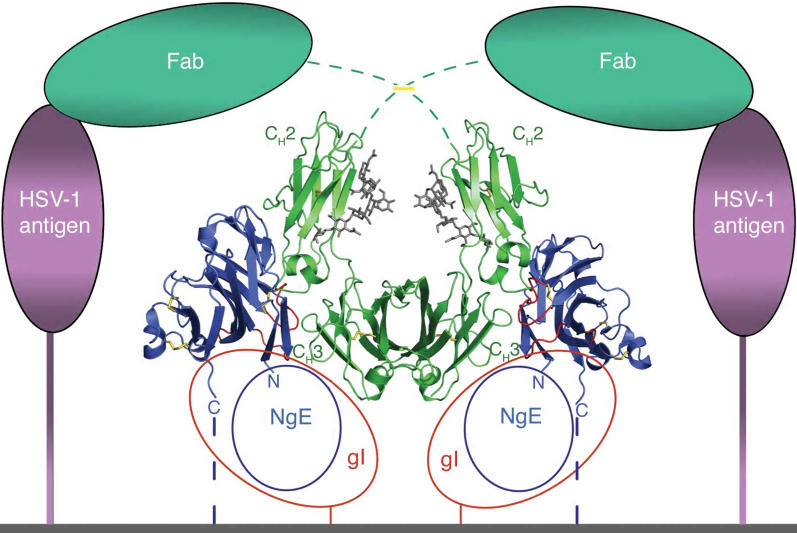
Model for gE-gI–Mediated Antibody Bipolar Bridging on the Cell Membrane A ribbon diagram of the CgE/Fc portion of the gE-gI/Fc structure is shown with the approximate positions of the gI chain (red open oval) and NgE domain (blue open oval) shown schematically. The predicted position of the cell membrane (see text) is indicated by a gray line. The upright position of the Fc dimer suggests that the hinge region (green dashed line) and Fab arms (green ovals) of an intact antibody are located above gE-gI, where the Fab combining sites could bind to a viral antigen (purple ovals) tethered to the same membrane as gE-gI.

Two studies used insertion mutagenesis to localize the IgG-binding site on gE [ 14, 17]. Five of 15 insertions that fall within the CgE domain were introduced into surface-exposed loops and are therefore unlikely to have global effects on the gE structure ( Figure 4B and 4C). Of these, three insertions (following gE positions 235, 348, and 380) are located on the top surface of CgE where they could interfere sterically with interactions between CgE and the C _H_1-C _H_2 linker and/or Fab domains of a bound IgG ( Figures 4B and 5). The remaining two surface-exposed loop insertions (following gE positions 333 and 339) are either in or close to the CgE/Fc interface predicted by the gE-gI/Fc structure ( Figure 4B and 4C). Ten other insertions are more likely to have a global effect on gE folding rather than a specific effect on IgG binding. These include insertions into interior regions of CgE (those following gE positions 256, 264, 302, 324, 355, and 371) or insertions that change the spacing between the disulfide bonds in the third β-sheet of CgE (those following gE positions 277, 285, 291, and 297) ( Figure 4B).

Insertion mutants in gE were also tested for their effects on viral spread in cultured epithelial cells and HSV-1 infected mice [ 7, 17, 18, 38, 39]. Although the mechanism of viral spread is unknown, it has been hypothesized that the extracellular domains of gE-gI bind to receptors at cell junctions, allowing entry into adjacent cells [ 17, 40]. Five insertions in CgE affected viral spread as well as IgG binding, of which four insertion mutants (following gE positions 277, 291, 348, and 380) were able to form a heterodimeric complex with gI [ 17]. As discussed above, the structural effects of insertions after gE positions 277 and 291 are difficult to interpret due to their location near disulfide bonds in the third β-sheet of CgE. However, the insertions after gE positions 348 and 380 are less likely to disrupt the overall structure of gE, therefore a region of CgE encompassing these residues ( Figure 4B) may be involved in binding receptors required for cell-to-cell spread of HSV-1.

### A Model for the gE-gI/IgG Complex at the Cell Surface

The CgE/Fc model, taken together with the locations of introduced heavy atoms and SeMet residues, provides insight into the organization of the gE-gI/Fc ternary complex and its orientation on a cell membrane. The position of the C-terminus of CgE in the gE-gI/Fc complex structure, which is ˜30 amino acids before the predicted transmembrane domain, suggests that IgG bound by gE-gI is in an upright orientation with respect to the membrane ( Figure 5), similar to FcαRI-bound IgA [ 41], but opposite to the orientation predicted for antibodies bound by other cellular Fc receptors such as FcγRIII and FcɛRI [ 42– 44].

The locations of NgE and gI in the gE-gI/Fc complex can be approximated by an analysis of heavy-atom and SeMet sites that are not accounted for by the CgE and Fc models. The extra heavy-atom sites are located in a space that is bounded by the C _H_3 domains of Fc on the top, the presumed membrane location on the bottom, and symmetry-related CgE molecules on the sides ( Figure 3B). Six of the sites that are not accounted for by CgE or Fc, and thus attributed to NgE or gI, are related by the Fc dyad axis (i.e., two sets of three heavy-atom sites), consistent with the two-fold symmetry of the complex that results from the inherent symmetry of the Fc dimer. These results constrain NgE and the gI ectodomain to occupy a position below CgE in the CgE/Fc model as shown in Figure 3B, in agreement with the presence of extra density that is not accounted for by the models for CgE or Fc in experimental electron density maps (unpublished data). Given that NgE is covalently attached to the N-terminus of CgE, we predict that NgE and gI, which is tethered to the membrane and interacts with NgE [ 16], are both located below CgE, as depicted schematically in Figure 5. The assignment of the NgE and gI domains in the vicinity of the Fc C _H_3 domain is consistent with the suggestion that additional binding surfaces on Fc include C _H_3 domain residues 356 and 358 [ 45]. The same study reported that allotypic changes in residue 214 in the IgG1 C _H_1 domain affected binding by gE-gI, which might be explained if flexibility in the hinge region between the Fab arms and Fc allowed contact of C _H_1 with the CgE portion of gE-gI. The proposed positions of NgE and gI, together with the upright orientation of Fc on a cell membrane, would allow the Fab arms of an intact anti-HSV IgG to interact with HSV antigens on the surface of the same membrane in a bipolar bridging complex ( Figure 5).

## Discussion

Here we report the structures of CgE, an Fc-binding fragment of the HSV-1 Fc receptor gE-gI, and a gE-gI complex with Fc. Because we were able to solve the crystal structure of the CgE subunit of the gE-gI ectodomain at high resolution, the identification of CgE as a minimal Fc-binding domain ( Figure 1) made its structure useful for interpreting the low-resolution gE-gI/Fc complex structure. An independent prediction of the complex structure using the coordinates of the unbound CgE and Fc molecules revealed a close agreement with the experimentally determined gE-gI/Fc structure ( Figure 3C), demonstrating the power of new protein docking methods [ 33, 34], and their potential for facilitating difficult crystallographic problems.

The structure of the CgE domain of gE-gI represents a new variant of the Ig superfamily that is distinct from the structures of host FcRs and other known Fc-binding proteins ( Figure 2C and 2D). CgE contains two β-sheets with a Greek-key folding topology similar to the topology of Ig V domains, but includes an extra β-sheet that packs against the Ig V–like portion of CgE ( Figure 2A and 2B). Even within the region of CgE that resembles an Ig V domain, CgE has a different disulfide-bonding pattern and is longer and more open than others in the Ig superfamily.

As revealed in the low resolution crystal structure of a gE-gI/Fc complex, the CgE portion of gE-gI binds to Fc at the C _H_2-C _H_3 interdomain junction ( Figure 3A), consistent with previous studies of the gE-gI/Fc interaction [ 12, 21, 23] and allowing a comparison of the Fc-binding properties of CgE with other proteins that bind to the same site on Fc [ 19, 20, 27]. An analysis of the CgE/Fc interface predicts that the six residues that are the hallmark of the consensus binding site on Fc [ 19, 27] are also central to Fc binding by CgE ( Figure 4A). In addition, the predominantly nonpolar CgE/Fc interface includes predicted contacts that are chemically similar to the conserved interactions with Fc found in several other FcR/Fc interfaces [ 19, 20, 27].

As is also the case for FcRn binding to Fc, the gE-gI interaction with Fc shows a sharp pH-dependent affinity transition near neutral pH, but in the opposite direction: whereas FcRn binds IgG at acidic pH in intracellular vesicles but not at the slightly basic pH of the cell surface [ 46], gE-gI binds IgG only at neutral or slightly basic pH [ 12]. Our analysis of gE-gI/Fc affinity as a function of pH suggested that the pH-dependent binding was due to protonation of two residues, likely histidines, in Fc and/or gE [ 12]. As CgE also shows pH-dependent binding to Fc ( Figure 1), the CgE/Fc model derived from the gE-gI/Fc structure can be used to identify candidate residues involved in the pH-dependent interaction, which include four histidines in the CgE/Fc interface (His247 on gE and His310, His433, and His435 on Fc) ( Figure 4A). Interestingly, His310 and His435 are two of the three Fc histidines that have been identified as conferring a sharp pH-dependence to the FcRn/Fc interaction [ 27]. In the FcRn/Fc complex these histidines are buried in titratable salt bridges that are disrupted at neutral or basic pH [ 27], whereas the histidines in the CgE/Fc interface are buried in a hydrophobic environment that would be disrupted upon protonation of the histidines at acidic pH, resulting in the acquisition of a positive charge. The distinct roles proposed for these Fc histidines depending on the nature of the Fc-binding protein is an example of the versatility of the consensus binding site at the Fc C _H_2-C _H_3 interdomain junction.

The model for CgE binding to Fc derived from the crystal structures of CgE and a gE-gI/Fc complex can be used to gain insight into the regions of CgE that are implicated in cell-to-cell spread of HSV. Mapping of insertion mutations in gE that affect viral spread [ 17, 18, 39] on the CgE and gE-gI/Fc complex structures identifies a region of CgE that could interact with receptors for gE-gI ( Figure 4B). Although the structure-based interpretation of the insertion mutagenesis studies on CgE suggests that the surface of the protein involved in cell-to-cell spread of the virus is distant from the Fc-binding interface ( Figure 4B), it is currently unclear whether the cell-to-cell spread and IgG binding functions of gE-gI are correlated. Viruses with insertions in gE after positions 333 and 339, which are at or near the crystallographically observed CgE/Fc interface, are disrupted for IgG binding but spread normally, suggesting that IgG binding is not required for cell-to-cell spread [ 7, 17, 38]. Consistent with this possibility, cell-to-cell spread has been observed in the absence of IgG when extracellular spread is blocked by the presence of solid matrices. However, the extent of cell-to-cell spread is influenced by the presence of anti–HSV-1 IgGs that contain Fc regions that can bind to gE-gI [ 47], suggesting a role for antibody bipolar bridging. A positive correlation between cell-to-cell spread and antibody bipolar bridging could be explained by a mechanism involving increased endocytosis of gE-gI bound to anti-HSV/HSV antigen complexes, dissociation and degradation of anti-HSV IgG in acidic vesicles, and transport of gE-gI to the trans-Golgi network [ 48, 49], where it would be available for viral packaging [ 50].

The crystallographic data can be used to deduce a model for how IgG binds gE-gI on the surface of virions or infected cells, whereby CgE binds the consensus Fc-binding site on each half of the Fc dimer, and gI and the NgE are located underneath the CgE domain between the C _H_3 domains of Fc and the membrane ( Figure 5). Critical to interpretation of gE-gI recognition of IgG in vivo is the prediction that intact IgG is bound in an “upright” orientation, with the C _H_3 domains of the Fc closest to the membrane and the Fab arms furthest away. Similarly, FcαRI binds the IgA Fc in an upright orientation [ 41], whereas in other host FcR/Ig complexes of known structure (the FcγRIII/Fcγ and FcɛRI/Fcɛ complexes), the receptor-bound antibody is oriented with its Fabs closest to the membrane [ 42– 44]. The upright orientation of the Fc and the positioning of the bulk of gE-gI to the side and underneath the Fc leave sufficient space for the Fab arms of an anti-HSV IgG bound to gE-gI to interact with antigens using antibody bipolar bridging ( Figure 5).

The gE-gI binding site on Fc does not directly overlap with the binding sites for the FcγRs or the C1q component of complement, which bind on or near the C _H_2 domain [ 43, 44, 51], thus the structure of the gE-gI/Fc complex does not directly suggest how gE-gI binding to the Fc region of IgG leads to evasion of FcγR- and complement-mediated immune responses. However, an anti-HSV antibody that is bound to both gE-gI and an HSV antigen on the surface of an infected cell could be sterically hindered from also binding to host FcγRs or C1q due to the close proximity of the proteins in the antibody bipolar bridging complex. Evasion of the host immune response may also be facilitated by the pH sensitivity of the gE-gI/Fc interaction [ 12]. The demonstration that the gE-gI/Fc structure is compatible with antibody bipolar bridging ( Figure 5) raises the possibility that anti-HSV IgG/HSV antigen complexes interacting with gE-gI on the surface of infected cells are endocytosed by gE-gI and degraded in the lysosomes after dissociation at acidic pH, resulting in destruction of antiviral antibodies and removal of viral antigens from the cell surface.

## Materials and Methods

### Purification and expression of CgE.

Our numbering scheme defines the first residue of the hydrophobic leader peptide in the immature gE protein as residue 1. N-terminal sequencing and mass spectroscopy analyses of degradation products present in a preparation of purified gE-gI suggested that gE residues 210–395 comprise a stable domain (unpublished data) in agreement with previous proteolytic analyses of soluble gE-gI that showed that gE was cleaved into N- and C-terminal fragments with a domain boundary in the vicinity of gE residue 208 [ 16]. Standard PCR-based subcloning techniques were used to insert a fragment of the gE gene from HSV strain KOS, encoding residues 213–390, downstream of the p10 promoter in the pAcUW51 baculovirus transfer vector (PharMingen, San Diego, California, United States). The expression vector also encoded the gE hydrophobic leader peptide N-terminal to the inserted fragment and a C-terminal Factor Xa cleavage site and 6x-His tag. Recombinant baculovirus stocks were generated by cationic liposome cotransfection of the expression plasmid with linear wild-type baculovirus DNA in High 5 insect cells (Invitrogen, Carlsbad, California, United States) as described by the manufacturer. Insect cell supernatants containing secreted CgE were buffer-exchanged into nickel-binding buffer (40 mM Tris [pH 8], 300 mM NaCl, 10 mM imidazole) and passed over a Ni-NTA agarose column (Qiagen, Valencia, California, United States). Bound protein was eluted in buffer containing 40 mM Tris (pH 8), 300 mM NaCl, and 250 mM imidazole, and further purified by size-exclusion chromatography on a Superdex 75 HiLoad 16/60 column (GE Healthcare, Piscataway, New Jersey, United States) that was equilibrated in 10 mM HEPES (pH 7.6) and 150 mM NaCl. Peak fractions were concentrated and buffer exchanged into 10 mM HEPES (pH 7.5). The expression of SeMet-substituted CgE was performed using an adaptation of published protocols [ 52, 53]. Briefly, insect cells that were infected with the CgE-recombinant virus were grown in Ex-Cell 400 media (JRH Biosciences, Lenexa, Kansas, United States) for 16 hours, pelleted, and then resuspended in Sf-900 II SFM media (Invitrogen) supplemented with 30 mg/l of L-cysteine (Sigma, St. Louis, Missouri, United States). After 4 h, 100 mg/l SeMet (Sigma) was added, and supernatants were harvested after 28 h and processed as described above for native CgE.

### Biosensor studies of CgE/Fc binding.

Surface plasmon resonance studies were performed using a BIAcore 2000 instrument (Biacore, Uppsala, Sweden). In this system, binding between a molecule coupled to a biosensor chip (the “ligand”) and a second molecule injected over the chip (the “analyte”) results in changes in the surface plasmon resonance signal that are read out in real time as resonance units [ 54]. wtFc and nbFc, both derived from human IgG1, were purified from CHO cell supernatants as described previously [ 12] and immobilized on a CM5 biosensor chip (Biacore) using primary-amine coupling as described by the manufacturer. Equilibrium binding data for CgE binding to wtFc (coupling density, 440 resonance units), nbFc (coupling density, 405 resonance units), and a mock-coupled flow cell were collected for a CgE concentration series (three-fold dilutions of CgE from 30 μM to 5 nM) at pH 8 (50 mM HEPES [pH 8.0], 150 mM NaCl, 3 mM EDTA, 0.005% [vol/vol] P-20 surfactant) and pH 6 (50 mM sodium phosphate [pH 6.0], 150 mM NaCl, 3 mM EDTA, 0.005% [vol/vol] P-20 surfactant). After each injection of CgE, a 30-s injection of 250 mM di-ammonium hydrogen citrate (pH 5.0) was used to disrupt the interaction and restore the surface.

Sensorgrams were processed and analyzed using the Scrubber software package (Biologic Software Pty. Ltd., http://www.biologic.com.au). K _D_ values were determined by plotting the equilibrium response in the plateau region versus the logarithm of the CgE concentration for each injection and fitting the data to a single-site binding model. Although Fc contains two potential binding sites for CgE, the interaction was too weak to accurately fit the data to a two-site binding model.

### Crystallization and structure determination of CgE.

Crystals of CgE were grown by hanging-drop vapor diffusion in drops containing equal volumes of ˜25 mg/ml CgE and well solution (100 mM Tris [pH 8.5], 28%–32 % [wt/vol] PEG 4000). Microseeding increased the frequency of obtaining single crystals. SeMet-substituted CgE crystals were grown in conditions similar to the native crystals with the addition of 10 mM MgCl _2_ in the well solution, which decreased the disorder that was often observed in the diffraction of the native crystals. All crystals were cryoprotected in well solution with an additional 15% PEG 4000, added in 5% increments, and stored in liquid nitrogen prior to data collection at −180 °C. Native and SeMet data were collected to 1.78 Å and 2.0 Å, respectively ( Table S1). Data were processed and scaled with the HKL suite (HKL Research, Charlottesville, Virginia, United States) [ 55], and the space group was determined as P2 _1_ with 2 molecules in the asymmetric unit.

Phases were derived from a three-wavelength multiple anomalous dispersion experiment using SeMet-substituted CgE crystals. Solve [ 56] was used for local scaling of the data, location and refinement of Se positions, and phasing (figure of merit of 0.36 from 30–2 Å) followed by Resolve [ 57, 58] for solvent flattening and automated model building (60% of the asymmetric unit was built by Resolve [ 57, 58]). The remainder of the model was built using O [ 59]. Multiple rounds of refinement, which included a bulk-solvent correction and positional refinement, simulated annealing with torsion angle dynamics, and individual B-factor refinement, were carried out with CNS [ 60] using the MLHL target with the SeMet data and phases and then switching to the MLF target with the native data. The final model was refined to 1.78 Å (R _cryst_ = 19.8%, R _free_ = 22.0%) and includes gE residues 218–390, residues 1–5 (first molecule) and 1–6 (second molecule) of the C-terminal Factor Xa recognition site, and 275 water molecules ( Table S1). A Ramachandran plot calculated using Procheck [ 61] showed that for nonglycine residues 87.1% are in the core regions, 12.2% are in the allowed regions, 0.6% are in the generously allowed regions, and 0% are in the disallowed regions.

### Crystallization and structure solution of a gE-gI/Fc complex.

Various forms of gE and the gE-gI heterodimer were subcloned, expressed, and purified from baculovirus-infected insect cell supernatants by nickel affinity and/or IgG affinity and gel-filtration chromatography as described previously [ 12]. Two recombinant forms of the Fc fragment of IgG1, wtFc and heterodimeric Fc, which contain two and one gE-gI binding sites, respectively, were also produced in CHO cells and purified as described previously [ 12]. Crystallization trials were conducted for various forms of CgE, gE, and gE-gI (including CgE [residues 213–390], gE [residues 21–419], gE2 [residues 21–390], gE-gI [gE plus gI residues 21–266], gE2-gI2A [gE2 plus gI residues 21–208], and gE2-gI2B [gE2 plus gI residues 21–201]) both alone and complexed with wtFc or heterodimeric Fc. The only isolated protein to crystallize was CgE (described above), and the only complex of the six possible gE-gI/Fc complexes that crystallized was one that contained gE residues 21–419 and gI residues 21–266 and wtFc (residues 223–447). The complex crystals grew from drops containing a 2:1 molar ratio of gE-gI and wtFc mixed with an equal volume of well solution (0.1 M MES [pH 6.0] or 0.1 M HEPES [pH 7.0] and 0.9–1.1 M sodium malonate), resulting in a final pH of approximately 7.5. Microseeding increased the reproducibility of crystal growth.

Complex crystals were prepared for data collection by transferring into drops containing either increasing concentrations of sodium malonate in 0.2 M increments up to 2 M, or 1.4 M sodium malonate plus increasing concentrations of glycerol in 5% (vol/vol) increments up to 15% and then stored in liquid nitrogen prior to data collection at −180 °C. Hundreds of crystals were screened for diffraction using synchrotron radiation at Stanford Synchrotron Radiation Laboratory beamlines 9–1 and 9–2. The best crystal diffracted anisotropically to ˜4.2 Å along the *l* axis and ˜5.4 Å along the *h* and *k* axes. Complex crystals were also screened for the effects of various cryoprotectants, crystallization additives, enzymatic deglycosylation of *N*-linked and *O*-linked sugar moieties on gE-gI, dehydration conditions, and annealing protocols on the diffraction; however, none of these variables significantly improved the quality of the diffraction.

A native dataset, which was collected on a complex crystal containing gE-gI that had been treated with *O*-glycosidase (Roche Applied Science, Indianapolis, Indiana, United States), was processed and scaled to 5.0 Å in the space group P4 _1_2 _1_2/P4 _3_2 _1_2 with the HKL suite [ 55] and MOSFLM/Scala [ 62] ( Table S2). Based on average volume to mass ratio of protein crystals [ 32], the asymmetric unit contained either one, two, or three copies of the 2:1 gE-gI/Fc complex, corresponding to 79%, 58%, or 37% solvent content, respectively.

A molecular replacement search was performed to 5.4 Å using the program Phaser [ 63, 64]. The search models were the CgE structure (reported in this paper) truncated after residue 390 (the last residue in gE before the Factor Xa cleavage site) and the human IgG Fc structure (pdb entry 1dn2), which together account for 45% of the total molecular mass of the gE-gI/Fc complex. Initial molecular replacement trials with the Fc coordinates clearly distinguished between the enantiomeric space groups P4 _1_2 _1_2 and P4 _3_2 _1_2 (log-likelihood gain of 44.7 for P4 _1_2 _1_2 versus 92.6 for P4 _3_2 _1_2), with P4 _3_2 _1_2 being the correct space group. Subsequent searches for CgE molecules, which were carried out in the space group P4 _3_2 _1_2 with the position of Fc fixed, yielded a log-likelihood gain of 282 for the first CgE and 578 for the second CgE. The positions of the first and second CgE molecules on each Fc chain were almost perfectly related by the dyad axis of the Fc, thus the CgE/Fc interface on each half complex is similar, with a slight translational shift of ˜2 Å for the second CgE molecule relative to the first. Because the asymmetric unit could contain one, two, or three 2:1 complexes, attempts were made to locate additional complexes, including searching for additional Fc molecules, searching for half complexes of one CgE and one Fc chain, and searching for additional 2:1 complexes. However, no additional CgE/Fc complexes were located, suggesting that the asymmetric unit contains one 2:1 CgE/Fc complex and ˜79% solvent.

For heavy-atom screening, complex crystals were soaked in 38 different heavy-atom compounds for between 18 h to 7 d, back-soaked for 2–3 h during cryopreservation, and screened for diffraction and fluorescence at the appropriate energies. Four datasets, which were collected near the *f′′* peak energies for crystals that were soaked in KIrCl _3_, PIP, EMP, or Na _2_WO _4_, were isomorphous with the native data set ( Table S2). All of the derivative data sets were indexed, reduced and scaled with MOSFLM, Scala, and Scaleit [ 62], respectively, before being input into Solve [ 56] with the native data set. Using the KIrCl _3_ and native data sets, Solve [ 56] initially identified five Ir atoms, whose locations mapped onto or near the position of the Fc molecule predicted by molecular replacement, that were used to calculate phases. Cross-difference Fourier calculations were performed for the PIP, EMP, and Na _2_WO _4_ derivatives, using the KIrCl _3_ phases to locate the heavy-atom binding sites for each derivative. Electron density maps were calculated using phases that were determined by combining all of the data sets in Solve [ 56] (figure of merit of 0.42 from 45–5 Å).

SeMet-substituted gE-gI was expressed in baculovirus insect cells as described above for CgE and purified as described above for native gE-gI. Crystallization and cryopreservation for crystals of the SeMet-substituted gE-gI/Fc complex were as described above for the native complex crystals. Multiple anomalous dispersion data were collected at the peak, inflection, and remote energies and processed using MOSFLM and Scala [ 62] ( Table S2). The SeMet data for each of the three wavelengths was input into Solve [ 56] with the KIrCl _3_ or four-derivative multiple isomorphous replacement with anomalous scattering phases, resulting in 13 Se sites (of a possible 18). Analysis of the sites reveal that eight Se positions map close to methionines in the CgE molecules as positioned in the molecular replacement solution, and 12 of the 13 sites are related by the Fc dyad axis (i.e., two sets of six sites). Phases determined in Solve [ 56] using only the 13 Se sites had a figure of merit of 0.41 from 30–6 Å. Residues in the interface are defined as those within 5 Å of a side-chain atom on the opposing protein.

### Theoretical prediction of the CgE/Fc interaction.

Prediction of the CgE/Fc interaction was done using the RosettaDock protocol as described [ 34]. Out of the initial population of 903,800 models generated in global docking calculations, the 200 lowest energy models that included at least one of the six “hot-spot” residues [ 19] at the predicted interface were clustered, and the clusters were ranked by the cluster size (the number of models within each cluster). In the next stage, a second constraint was imposed requiring that CgE make contact with both the C _H_2 and C _H_3 domains of the Fc protein, as observed for other proteins binding to the Fc “hot spot,” [ 19] which resulted in pruning some of the top-ranking clusters. The centers of each of the top five remaining clusters were then subjected to more extensive local rigid-body and side-chain refinement to produce five final predicted structures, of which one matches the crystallographic model of the CgE/Fc interaction ( Figure 3C) (model “d,” described below). In the five RosettaDock-predicted CgE/Fc complexes, different regions of CgE are used to bind the consensus region of Fc (model “b,” CgE residues in strand A, A-A′ linker, strand A′, strand B′, CI–CI′ linker, strand G, G–I′′ linker, and strand I′′; model “c,” CgE residues in strand A, A–A′ linker, B–B′ linker, strand B′, CI–CI′ linker, C′–C′′ linker, strand G, and strand I′′; model “d,” which resembles the molecular replacement solution, CgE residues in strand A, B–B′ linker, strand B′, strand C, strand C′, C′–C′′ linker, and D–E linker; model “e,” CgE residues in CI′–C′ linker, strand C′′, C′′–D linker, strand D, and E–F linker; and model “f,” CgE residues in strand C, C–CI linker, CI–CI′ linker, strand C′, and strand C′′, C′′–D linker, E–F linker) (unpublished data).

## Supporting Information

Figure S1 Comparison of CgE with Its Closest Structural Homologs Stereo superposition of the Cα atoms of (A) CgE (blue) with an Fv heavy chain (magenta; pdb entry 1mfa), and (B) CgE (blue) with coxsackie virus and adenovirus receptor domain 1 (green; pdb entry 1eaj). Disulfide bonds are in yellow.
(2.8 MB TIF)

Figure S2 Experimental Electron Density Map The molecular replacement solution is shown as a Cα trace superimposed on the experimentally phased electron density map. CgE is in blue with the CDR loops colored red, and Fc is in green with the carbohydrates in gray. A stereo view of the electron density within 5 Å of the molecular replacement model is shown for (A) Fc and (B) the CgE/Fc interface.
(6.7 MB TIF)

Table S1 Data Collection and Refinement Statistics of CgE (74 KB DOC)

Table S2 Data Collection and Phasing Statistics of a gE-gI/Fc Complex (74 KB DOC)

### Accession Numbers

The Research Collaboratory for Structural Bioinformatics Protein Data Bank accession numbers ( http://www.rcsb.org/pdb) are 2GIY for the CgE structure and 2GJ7 for the gE-gI/Fc complex structure.

## Abbreviations

CDR: complementarity-determining region
CgE: C-terminal domain of the gE ectodomain
FcR: Fc receptor
HSV-1: herpes simplex virus type-1
IgG: immunoglobulin G
nbFc: nonbinding Fc mutant
NgE: N-terminal domain of gE
pIgR: polymeric Ig receptor
SeMet: selenomethionine
V: variable
wtFc: wild-type Fc
