# Supplementary material for: Crystal Structure of the HSV-1 Fc Receptor Bound to Fc Reveals a Mechanism for Antibody Bipolar Bridging
Source: PLoS Biol. 2006 May 2;4(6):e148. doi: 10.1371/journal.pbio.0040148 (PMC1450327; doi:10.1371/journal.pbio.0040148)
Supplement: Supplementary file 1 [file pbio.0040148.st001.doc]

Table S1 (Sprague, et al.)

Table S1 Data collection and refinement statistics of CgE

| Data Collection |  | | | |
| --- | --- | --- | --- | --- |
|  |  |  |  |  |
| Data Set | Native | SeMet-peak | SeMet-inflection | SeMet-remote |
| Resolution (Å)^a^ | 1.78  (1.81-1.78) | 2.0  (2.03-2.00) | 2.0  (2.03-2.00) | 2.0  (2.03-2.00) |
| Cell constants (Å, º) | a=45.7, b=91.2, c=47.4,  β=117.6 | a=45.3, b=91.7, c=46.8,  β=117.6 | a=45.4, b=91.8, c=46.8,  β=117.6 | a=45.4, b=91.7, c=46.8,  β=117.6 |
| Wavelength (Å) | 0.97060 | 0.9788 | 0.9795 | 0.9050 |
| Measured reflections | 298,162 | 147,743 | 149,403 | 151,468 |
| Unique reflections | 33,227 | 22,997 | 22,906 | 22,911 |
| Mean I/ σ(I) | 19.2 (4.3) | 21.1 (3.5) | 21.8 (3.0) | 21.8 (3.4) |
| R_sym_ (%)^b^ | 8.2 (33.1) | 4.3 (25.7) | 4.2 (28.7) | 4.2 (26.6) |
| Completeness (%) | 98.6 (72.6) | 99.4 (99.1) | 99.4 (98.5) | 99.5 (99.1) |
| Redundancy | 5.0 (4.2) | 3.6 (3.3) | 3.6 (3.3) | 3.6 (3.3) |
|  |  |  |  |  |
| Refinement |  |  |  |  |
|  |  |  |  |  |
| Resolution (Å) | 30-1.78 | Ramachandran plot |  |  |
| Reflection used | 32,739 | % favored | 87.1 |  |
| Working set | 29,526 | % allowed | 12.2 |  |
| Test reflections | 3213 | % generous | 0.6 |  |
| Number of  nonhydrogen atoms  (protein/water) | 2741/275 | % disallowed | 0.0 |  |
| R_cryst_ (%)^c^ | 19.8 |  |  |  |
| R_free_ (%)^c^ | 22.0 |  |  |  |
| Average B factor | 19.3 |  |  |  |
| rmsd bond length (Å) | 0.007 |  |  |  |
| rmsd bond angle ( º) | 1.4 |  |  |  |

^a^ Values in parantheses are for the highest resolution shell.

^b^ R_sym_ = 100 × Σ⏐I - 〈I〉⎢ ⁄ ΣI, where I is the integrated intensity of a given reflection.

^c^ R_cryst_ = 100 × Σ⏐⎜F_obs_⎜- ⎜F_calc_⎜⎢ ⁄ Σ ⎜F_obs_⎜, where F_obs_ and F_calc_ are the observed and calculated structure factor amplitudes, respectively, for reflections in the working set. R_free_ was calculated for the test set containing randomly chosen reflections that were not included in the refinement.
