# Supplementary material for: Crystal Structure of the HSV-1 Fc Receptor Bound to Fc Reveals a Mechanism for Antibody Bipolar Bridging
Source: PLoS Biol. 2006 May 2;4(6):e148. doi: 10.1371/journal.pbio.0040148 (PMC1450327; doi:10.1371/journal.pbio.0040148)
Supplement: Supplementary file 2 [file pbio.0040148.st002.doc]

Table S2 (Sprague, et al.)

Table S2 Data collection and phasing statistics of a gE-gI/Fc complex

| Data Collection |  | | | | | | | |
| --- | --- | --- | --- | --- | --- | --- | --- | --- |
|  |  |  |  |  |  |  |  |  |
| Data Set | Native | KIrCl_3_ | PIP | EMP | Na_2_WO_4_ | SeMet-  peak | SeMet-  inflection | SeMet-  remote |
| Resolution (Å)^a^ | 5.0  (5.27-5.00) | 6.0  (6.32-6.00) | 6.4  (6.75-6.40) | 5.0  (5.27-5.00) | 6.2  (6.54-6.20) | 5.0  (5.27-5.00) | 5.3  (5.59-5.30) | 5.5  (5.80-5.50) |
| Cell constants (Å, º) | a=b=174.9  c=316.3 | a=b=174.9  c=317.7 | a=b=178.0  c=318.1 | a=b=174.6  c=319.55 | a=b=175.9  c=318.0 | a=b=175.9  c=315.3 | a=b=176.6  c=315.9 | a=b=176.9  c=315.6 |
| Wavelength (Å) | 0.9537 | 1.1048 | 1.0717 | 1.0060 | 1.2140 | 0.9792 | 0.9794 | 0.9322 |
| Measured reflections | 451,557 | 163,322 | 194,061 | 199,450 | 147,562 | 169,929 | 178,770 | 184,932 |
| Unique reflections | 21,931 | 11,899 | 11,049 | 20,924 | 11,935 | 18,984 | 15,984 | 14,506 |
| Mean I/ σ(I) | 21.3 (4.2) | 19.4 (1.9) | 15.6 (2.1) | 14.7 (2.0) | 18.7 (2.9) | 14.1 (3.3) | 15.2 (3.1) | 16.2 (3.4) |
| R_sym_ (%)^b^ | 10.6 (70.8) | 5.5 (42.2) | 7.1 (46.3) | 5.3 (47.6) | 7.8 (47.6) | 7.5 (45.6) | 7.7 (46.5) | 6.6 (43.3) |
| Completeness (%) | 99.7 (100.0) | 93.4 (94.2) | 99.4 (100.0) | 95.9 (97.0) | 99.9 (100.0) | 87.2 (90.2) | 86.6 (89.2) | 87.6 (90.2) |
| Redundancy | 11.2 (10.6) | 3.4 (3.3) | 4.5 (4.6) | 3.4 (3.0) | 5.7 (5.9) | 4.1 (4.1) | 4.1 (4.1) | 4.2 (4.2) |
|  |  |  |  |  |  |  |  |  |
| Phasing |  |  |  |  |  |  |  |  |
|  |  |  |  |  |  |  |  |  |
| Number of sites |  | 6 | 5 | 2 | 4 |  |  |  |
| Phasing power^c^ (acentric/centric) |  | 1.31/0.70 | 1.19/0.77 | 0.50/0.79 | 0.84/0.72 |  |  |  |
| Mean figure of merit | 0.42 |  |  |  |  |  |  |  |

^a^ Values in parantheses are for the highest resolution shell.

^b^ R_sym_ = 100 × Σ⏐I - 〈I〉⎢ ⁄ ΣI, where I is the integrated intensity of a given reflection.

^c^ Phasing power = 〈F_H_〉|/〈E〉, where F_H_ is the heavy-atom structure factor amplitude and E is the residual lack-of-closure error.
